# Semi-Supervised Bidirectional Long Short-Term Memory and Conditional Random Fields Model for Named-Entity Recognition Using Embeddings from Language Models Representations

**DOI:** 10.3390/e22020252

**Published:** 2020-02-22

**Authors:** Min Zhang, Guohua Geng, Jing Chen

**Affiliations:** 1 School of Information Science and Technology, Northwest University, Xi’an 710127, China; zhangmin@stumail.nwu.edu.cn (M.Z.); chenjing@stumail.nwu.edu.cn (J.C.); 2 School of Engineering and Technology, Xi’an Fanyi University, 710105 Xi’an, China

**Keywords:** cultural relics, named-entity recognition, semi-supervised learning, embeddings from language models, bidirectional long short-term memory network, conditional random fields

## Abstract

Increasingly, popular online museums have significantly changed the way people acquire cultural knowledge. These online museums have been generating abundant amounts of cultural relics data. In recent years, researchers have used deep learning models that can automatically extract complex features and have rich representation capabilities to implement named-entity recognition (NER). However, the lack of labeled data in the field of cultural relics makes it difficult for deep learning models that rely on labeled data to achieve excellent performance. To address this problem, this paper proposes a semi-supervised deep learning model named SCRNER (Semi-supervised model for Cultural Relics’ Named Entity Recognition) that utilizes the bidirectional long short-term memory (BiLSTM) and conditional random fields (CRF) model trained by seldom labeled data and abundant unlabeled data to attain an effective performance. To satisfy the semi-supervised sample selection, we propose a repeat-labeled (relabeled) strategy to select samples of high confidence to enlarge the training set iteratively. In addition, we use embeddings from language model (ELMo) representations to dynamically acquire word representations as the input of the model to solve the problem of the blurred boundaries of cultural objects and Chinese characteristics of texts in the field of cultural relics. Experimental results demonstrate that our proposed model, trained on limited labeled data, achieves an effective performance in the task of named entity recognition of cultural relics.

## 1. Introduction

The internet is changing people’s lives and the manner in which cultural relics are displayed [1]. With the development of smart museums and digital museums, online museums have drawn considerable attention in the field of cultural relics. A growing number of researchers are engaged in online information extraction of cultural relics. The online information of museums can make cultural relics come alive and provide data sources for the protection of cultural relics and the retrieval of knowledge graphs of cultural relics [2,3]. The first step for extracting potential knowledge automatically from the vast amounts of online cultural relics information is named-entity recognition (NER), which is an important part of information extraction and knowledge graphs [4,5].

In this study, our aim is recognizing several types of entities of cultural relics, including cultural relics’ name (CRN), cultural relics’ dynasty (CRD), unearthed location (UL), and museum collection (MC), which are critical concepts in the knowledge discovery of cultural relics. Cultural relics’ name entity recognition aims to extract the name of cultural relics in text. The cultural relics’ dynasty entity recognition task seeks to find the dynasty of cultural relics, that is, the time when the relics were made. The unearthed location entity recognition task attempts to locate the unearthed location of cultural relics. The museum collection entity recognition task records the museum where the cultural relics’ collection resides.

For example, “*In 1977, Three Sheep Bronze Lei (Lei is an ancient urn-shaped wooden wine vessel) was unearthed in Liu Jiahe, Pinggu, Beijing. ……The Sheep head of the Lei utilized the step casting method, which revealed the height level of the Shang dynasty bronze casting technology. It is now in the capital museum*”. In this post, “*Three Sheep Bronze Lei*” is the cultural relic name entity, “*Liu Jiahe, Pinggu, Beijing*” is the unearthed location entity, “*Shang dynasty*” is the cultural relic dynasty entity, and “*the capital museum*” is the museum collection entity. 

Modern neural models for NER depend upon word representations, which may be based on words, characters, or any combination of words and characters. In the past ten years, pretrained word embeddings have been widely used as features and improved according to the characteristics of the corpus in the NER task. However, the features contained in a character are not being used effectively. Some researchers utilize character embedding to address this problem [6]. Nevertheless, the use of character embedding alone will result in the absence of the characteristics of the relationship between words. Then, the combination of word embedding and character embedding is applied to the NER task. Regrettably, the combination does not make good use of contextual characteristics to improve the performance of NER [7]. 

In the task of cultural relics NER, due to the word formation, particularity of the cultural relics’ named entity, the same word has different meanings in different applications. For example, “*杜虎符: Bronze Tiger-shaped Tally*” is the name of the cultural relic, and tiger is an animal. Therefore, it is essential to obtain contextual information. Recently, Peters et al. [8] introduced a novel deep contextualized word representation model named Embedding from Language Model (ELMo). Moreover, a particularity of syntactic structure and semantics exists in the Chinese corpus. ELMo solves this problem well and can better obtain the syntactic and semantic features of Chinese context. Therefore, we apply ELMo to generate the word representations to obtain an effective NER result in our task.

As a key component in the field of information extraction, NER has been invested with continuous attention for decades. The traditional machine learning approaches, such as conditional random fields [9] and maximum entropy [10], have been utilized in the NER tasks in past years. Conditional random fields (CRFs) have been proven to be effective in many areas of natural language processing (NLP), including sequence tag tasks and named-entity recognition (NER). Compared to the other statistical models (e.g., ME), the advantage of CRFs is that an observation sequence with a large number of features is utilized in CRFs. CRFs depend on hand-crafted features and domain-specific knowledge extracted for a special domain in NER tasks. Nevertheless, hand-crafted features are difficult to develop. Neural networks, particularly LSTMs, have recently been shown to be effective for NER tasks. LSTMs enable the automatic leveraging of orthographic features and avoid extracting features manually when performing NER tasks. However, it is difficult for the LSTM model to learn the complete markup rules due to the lack of training data, and it is impossible to perform optimization processing for global sequences such as CRFs. Many studies have shown that a combination of different learning systems (LSTM and CRF) is a better method to obtain excellent performance. The combination of LSTM and CRF models can not only solve the problem of obtaining hand-crafted features but also effectively tag the sequence. Therefore, we utilize the combination of LSTM and CRF models to complete our NER task, which is similar to the framework proposed by Yang et al. [11].

Recently, deep learning has emerged as an outstanding application for the NER task. Instead of depending on hand-crafted data, deep learning methods can automatically extract complex features, which have a richer representation ability [12]. Despite such attractiveness, deep learning approaches always depend on large amounts of high-quality labeled data to promote NER performance. In many practical applications, the labeled data are quite limited and attained uneasily, mainly due to the time-consuming and burdensome expense of manual annotation. Moreover, a considerable amount of unlabeled data are easily available. Therefore, it is indispensable to explore an effective method for a training framework to address the problem of lacking labeled data in NER tasks. Self-labeled is a commonly suitable semi-supervised method which solve the scarce of labeled data through a self-learning process based on supervised prediction models [13]. In the three algorithms (self-training, co-training and tri-training) of the self-labeled method, the self-training algorithm is a simple, efficient and commonly self-labeled methods [14,15]. Inspired by the successful application of semi-supervised learning and the self-training method, we apply self-training of a semi-supervised method using both labeled and unlabeled data to improve the performance of NER for cultural relics. 

Although Chinese entity recognition has been widely studied, existing approaches have several challenges when utilizing Chinese social media data. First, morphological changes seldom exist in Chinese. Second, Chinese provides very scant information about word locations and boundaries (e.g., English named entities usually start with a capital letter). Third, compared with English, there is no blank space between words in Chinese, making existing entity extraction approaches challenging in the context of Chinese. In addition to the inherent particularity of the Chinese language, cultural relics data also have some challenges. For example, cultural relic entities are complex and diverse, and the boundaries between words are vague and difficult to define.

At the present stage, cultural relics entity recognition provides basic support for the retrieval of cultural relics and the construction of intelligent museums. However, the existing research is unsuitable for the Chinese social media context in the task of cultural relics’ entity extraction. Motivated by these observations, this paper proposes a model named semi-supervised cultural relics named-entity recognition (SCRNER) for cultural relics’ entity recognition using Chinese online cultural relics information. The SCRNER model utilizes the basic architecture = composed of the bidirectional long short-term memory (BiLSTM) and conditional random fields (CRF) and uses the structure of word embedding pretrained by ELMo to learn effective features to recognize cultural relic entities. We obtain data from the online museum, eliminate noise, and perform word segmentation and other processing. The experimental results show that our method has efficient performance in the cultural relics entity identification task. 

The goal of this paper is to construct a model to effectively identify the cultural relic named entities from the large amount of unlabeled data generated by the online museums using a small amount of hand-labeled data. The main contribution of our work can be summarized as follows:

- We propose a semi-supervised model named SCRNER which is composed by the bidirectional long short-term memory (BiLSTM) and conditional random fields (CRF) to recognize cultural relic entities;
- We propose a sample selection strategy named the relabeled strategy, which selects samples of high confidence iteratively, aiming to improve the performance of the proposed semi-supervised model with limited hand-labeled data;
- We pretrain the ELMo model to generate the context word embedding, which makes our proposed model capable of capturing not only the features of the focal character but also the contextual information of the related word.

The remaining content of this paper is presented as follows. Prior relevant works on embedding, models and semi-supervised approaches for NER are organized in Section 2. In Section 3, we introduce the method of our proposed cultural relic entities extraction. In Section 4, we describe the evaluation and experimental results, followed by a discussion in Section 5. Section 6 offers conclusions about our major research findings.

## 2. Related Work

### 2.1. Embeddings for NER

#### 2.1.1. Word-Level Embedding 

In the NER task, pretrained word embeddings are widely used as features and improved according to the characteristics of the corpus. The word-level multilayer neural network model was first proposed by Collobert et al. [16]. This model avoided task-specific engineering as much as possible and discovered the internal representations of words during model training. Their architecture was similar to our architecture of embeddings from language models (ELMO)-based BiLSTM-CRF model, but the BiLSTM was replaced by a convolution layer, and the input of the CRF layer was the output of the convolution layer for the final prediction. The results of the architecture showed significant performance improvements in the NER tasks. Wu et al. [17] explored two popular neural word-embedding algorithms. The results showed that the distributed word-embedding features achieved from the training of a large number of unlabeled corpora are better than those achieved by clustering. Neural word embedding captures semantic relationships, and the distributed representation of features are obtained by the discretization of these semantic relationships.

#### 2.1.2. Character-Level Embedding

Word embedding in Chinese needs to be trained after word segmentation in the original corpus. Additionally, some advanced systems of the Chinese NER may not use word embedding. 

Since Kim et al. [18] first used character-level embedding, many scholars have applied the potential of characters in NER neural models. Dong et al. [19] were the first to utilize the character-based neural architecture in Chinese NER. The evaluation results of the system showed that the system achieved a performance of 90.95% F1. Xu et al. [20] proposed a simple and effective neural model, named ME-CNER, which applied character-level embedding in the NER task. Rich semantic information is used to implement character embedding at multiple granularities. Their approach achieves a large performance improvement on the Chinese Weibo dataset.

#### 2.1.3. Character and Word-Level Embedding 

The NER systems that combine word context and word characteristics have been proven to be powerful because they require little domain knowledge [6]. Chen et al. [21] presented a character-enhanced word embedding (CWE) framework that jointly learned character and word embeddings to reduce excessive dependence on external information. The results of experiments showed that the quality of word embeddings was significantly improved by character embedding. Zeng et al. [22] offered a recurrent neural network based on the LSTM-CRF model that combined word embedding with the character-based representation. Experimental results achieved good performance in the DDI2013 challenge.

#### 2.1.4. ELMo Embeddings

In the ELMo model [8], the word vectors were learned from a deep bidirectional language model (biLM) pretrained on a large amount of text corpus. Word embedding that extracted the corresponding words from the pretrained network was a feature supplement. It has been shown that ELMo can improve the performance of the different NLP tasks.

Yang et al. [23] proposed a multitask attention-based biLSTM-CRF, named the Att-biLSTM-CRF model, which applied a pretrained ELMo. Their method developed the multitask mechanism to improve the recall of the clinical named-entity recognition. Straková [24] proposed two neural network architectures for nested NER, and the ELMo contextual embeddings were used to enrich their architectures. Dogan et al. [25] proposed a framework that incorporated deep learning models of ELMo with Wikidata to address the issue of the lack of datasets for the task of fine-grained NER. Their model learned representations based on context and combined the abundant structure of Wikidata to predict labels of finer-grained subtypes.

Word-level embedding, character-level embedding and character-level combined word-level embedding achieved some effective results in NER tasks, but they have trouble obtaining contextual characteristics. Accordingly, the particularity of syntactic structure and semantics exists in the Chinese corpus. ELMo solves this problem well and can better obtain the syntactic and semantic features of Chinese context. In our work, we use the pretrained ELMo to generate the word representation. 

### 2.2. Model for NER 

As a key component in the field of information extraction, named-entity recognition (NER) has been the focus of continuous attention for decades. The traditional machine learning approaches have been utilized in the NER tasks in recent years. 

Isozaki et al. [26] implemented NER tasks using support vector machines (SVMs) and proved that the model was more inefficient than the conventional model. At the same time, they implemented a faster classifier to speed up the efficiency of the system. Bender et al. [27] applied maximum entropy (ME) models to implement the NER tasks. First, they built an NE recognizer starting with a labeled dataset and a feature set. Then, the NE recognizer was applied to recognize the named entities.

#### 2.2.1. CRF Model for NER 

Conditional random fields (CRFS) have been proven to be effective in many areas of natural language processing (NLP), including sequence tag tasks and named-entity recognition (NER). Compared to the other statistical models (e.g., ME), the advantage of CRFs is that an observation sequence with a large number of features is utilized in CRFs.

Chen et al. [28] presented a Chinese NER system based on conditional random fields (CRFs), which combined the basic features and the additional features acquired from evaluating statistics in the training corpus. They applied the n-best results produced by the CRF model to perform the postprocessing to correct inconsistent results. Sobhana et al. [29] developed a system for NER tasks of geological text based on conditional random fields (CRFs), which applied the contextual information of words and the word features to predict the various named-entity (NE) classes. 

#### 2.2.2. LSTM Model for NER 

CRFs depend on hand-crafted features and domain-specific knowledge extracted for a special domain in NER tasks. Nevertheless, hand-crafted features are difficult to develop. Neural networks, particularly LSTMs, have recently been shown to be effective for NER tasks. The LSTM enables the automatic leveraging of orthographic features and avoids extracting features manually when performing NER tasks. 

Limsopatham and Collier [30] investigated an approach for NER in Twitter messages. In this approach, the LSTM model was enabled to tackle the problem of the short, noisy and colloquial nature of tweets. Compared with other systems, their system achieved the most effective performance. Hammerton [31] proposed an approach that applied the LSTM to complete the task of named-entity recognition. The model was trained to run two passes on each sentence, and the tags of the decisions were output on the second pass. 

#### 2.2.3. BiLSTM_CRF Model for NER

Many studies have shown that the combination of different learning systems is a better method to obtain excellent performance. 

Huang et al. [32] proposed a BILSTM-CRF model that obtained context features through a bidirectional LSTM and utilized sentence-level tag information through the CRF. The model was robust and produced higher accuracy compared to previous observations. Accordingly, their model obtained accurate tagging performance and depended less on word embedding. Other domains applied similar systems, such as medical NER by Kai [33]. Their model was based on bidirectional LSTM and CRF, named BiLSTM-CRF, which contained three layers and applied the character-level word representations trained from the supervised dataset to learn the characteristics. Experiments have shown that their approach outperformed the baseline methods. Lample et al. [34] provided a hybrid tagging architecture based on LSTMs and CRFs, which was similar to the approach presented by Huang et al. Their models depended on character-level word representations trained from the labeled corpus and unsupervised word representations trained from the unlabeled corpus.

In this paper, we propose a model-based BiLSTM that can use both past and future input features, and a CRF layer which can use sentence-level tag information. Experiments show that the BiLSTM-CRF model can produce accurate tagging performance in our task of cultural relics NER.

### 2.3. Semi-Supervised Learning for NER

In the task of NER, the labeled data are not sufficient but massive unlabeled data are subsistent in many domains. To reduce dependence on labeled data and take advantage of large amounts of unlabeled data, many semi-supervised deep learning methods are applied to various domains of NER. Ji H, Grishman [35] proposed using the relevant measures of similar information retrievals to select documents and improve the existing NE classifier to address the problem of unlabeled data selection. Xu et al. [36] proposed a unified model, in which semi-supervised learning can learn in-domain unlabeled information by self-training. NER performance is improved by combining large amounts of unlabeled data. Liao et al. [37] presented a semi-supervised learning algorithm for NER using CRFs. Their algorithm exploited independent evidence to provide high-precision labels for unlabeled data. This independent evidence can automatically extract data with high accuracy and no redundancy. Then, an improved classifier would be the result in the next iteration. Liu and Zhang et al. [38] proposed a semi-supervised learning framework combining the K-nearest neighbors (KNN) classifier and the linear conditional random fields (CRF). The semi-supervised learning and gazetteer addressed the problem of insufficient training data. Luan et al. [39] proposed a semi-supervised algorithm that extended self-training based on the graph along with a data selection strategy to leverage unlabeled data.

Some progress has been made in the application of semi-supervised NER. Due to the small amount of data in the field of cultural relics, in order to improve the NER performance in the field of cultural relics, we propose a semi-supervised depth model to improve the performance of NER in the field of cultural relics.

## 3. Methods

**Figure 1:**
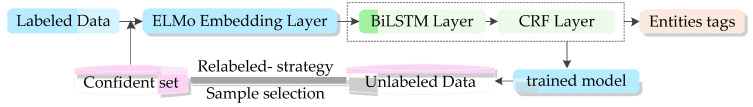
Overview of the semi-supervised bidirectional long short-term memory (BiLSTM)-conditional random fields (CRF) framework for named-entity recognition (NER).

In this paper, we propose a semi-supervised model named SCRNER to extract cultural relic entities from Chinese social media and the Chinese online museum based on the BiLSTM-CRF model. The model mainly includes two stages: training the BiLSTM-CRF model and updating the labeled data. An overview of our approach is shown in Figure 1.

In the first stage, a small amount of labeled data is input into the pretrained language model and uses ELMo to generate the word embedding. Then, the trained word embeddings are applied to train the BiLSTM-CRF model. After that, a large amount of unlabeled data is fed into the trained classifier to predict the labels. In the second stage, the relabeled-strategy sample selection method is utilized to select the samples of high confidence and form the confident set. Then, the confident set will be applied to train the next round of the model, and the classifier will be improved at the next iteration. The two stages of training the BiLSTM-CRF model and updating the labeled data repeat iteratively until the stopping criterion is reached. Through iterative training of the semi-supervised model, we obtain a large number of data with high confidence to train the BiLSTM-CRF model, and finally obtain the entity tag of the test data through this model.

### 3.1. ELMo Contextual Word Embedding 

ELMo is a new type of deep contextualized word representation that can model complex features (such as syntax and semantics) and changes of words in the language context (i.e., modeling polysemy). ELMo can generate different word vectors for the same word in different sentences, which more effectively solves the problem of polysemy. Due to the word formation particularity of the cultural relic named entities, we used ELMo to generate word representations.

The main method of ELMO is training a complete language model, and then using this language model to deal with the need to train text and generate the corresponding word embedding. When we use word embedding, the word has a specific context; at this time, word embedding can be fine-tuned according to the context semantics. The fine-tuned word embedding can better express the specific meaning of this context and solve the polysemy problem. Therefore, ELMO is essentially the process of dynamically adjusting word embedding according to the current context. 

**Figure 2:**
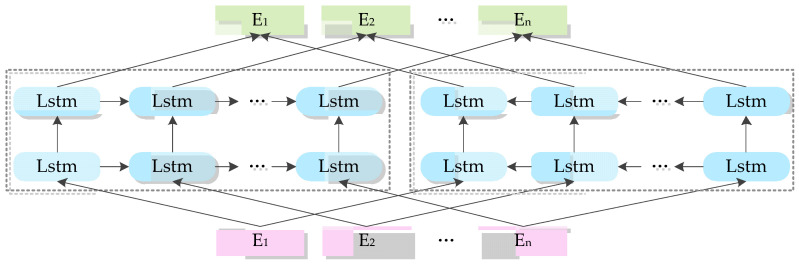
Embedding from Language Model.

The structure of the language model is shown in Figure 2.

Embedding from Language Model (ELMo), based on the bidirectional LSTM (biLSTM) language model, is a pretrained deep contextualized word embedding model. Each word token has its own embedding even though it has the same word type and the embedding of word tokens also depends on its context. The prediction formulation of the biLSTM language model, which is trained from many sentences, is to jointly maximize the log likelihood of the probability of token k from the forward and backward directions

(1) $$\overset{N}{\underset{k=1}{\sum }}(log\;p(t_{k}|t_{1}\ldots ,t_{k-1};\Theta_{x},{\overset{\leftarrow }{\Theta }}_{LSTM},\Theta_{s}+log\;p(t_{k}|t_{k+1}\ldots ,t_{N};\Theta_{x},{\overset{\leftarrow }{\Theta }}_{LSTM},\Theta_{s})),$$

where $t_{k}$ is token $k,\;t_{1}\ldots ,t_{k-1}$ and $t_{k+1}\ldots ,t_{N}$ represent the forward context and backward context of token *k*, respectively. $\Theta_{x}$ and $\Theta_{s}$, maintaining separate parameters for the forward and backward direction of the LSTMs, are the parameters for the token representation and Softmax layer, respectively. ${\overset{\leftarrow }{\Theta }}_{LSTM}$ and ${\overset{\leftarrow }{\Theta }}_{LSTM}$ represent the parameters of the LSTM in each direction.

Then, ELMo combines each biLSTM layer representations of token k as follows

(2) $$R_{k}=\left\{X_{k}^{LSTM},{\vec{h}}_{k,j}^{LSTM},{\overset{\leftarrow }{h}}_{k,j}^{LSTM}|j=1,\ldots ,L\right\}=\left\{h_{k,j}^{LSTM}|j=1,\ldots ,L\right\},$$

where $R_{k}$ represents the representation of token k, and $h_{k,j}^{LSTM}$ equal to [${\vec{h}}_{k,j}^{LSTM},{\overset{\leftarrow }{h}}_{k,j}^{LSTM}$] is for each layer of biLSTM.

ELMo collapses all the representations of each layer in R into one single vector. More generally, the top layer is selected by ELMo and a specific weighting of all biLSTM layers is computed by

(3) $$ELMo_{k}^{task}=\gamma^{task}\overset{L}{\underset{j=0}{\sum }}s_{j}^{task}h_{k,j}^{LSTM},$$

where $\gamma^{task}$ represents the scalar parameter, which is used to scale the ELMo vector on the basis of the feature of the task. $s_{j}^{task}$ is the softmax-normalized weight of each layer. 

### 3.2. Neural Network Architecture

**Figure 3:**
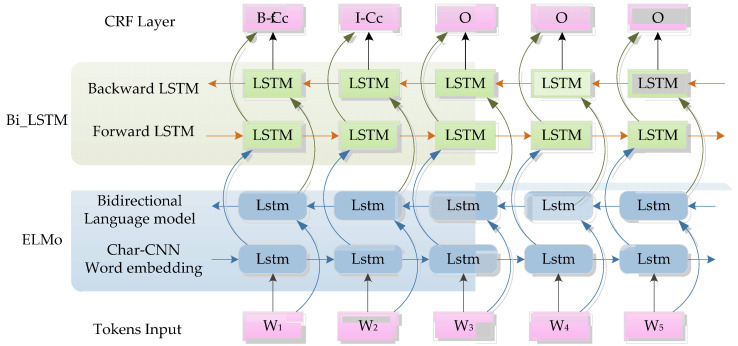
The architecture of embeddings from language models (ELMO)-based BiLSTM-CRF model.

The ELMo-based BiLSTM-CRF architecture is shown in Figure 3. A list of tokens is the input and the predicted entity types are the output of the model. The pretrained ELMo, together with a residual LSTM, are used to learn informative morphological representations from the character sequence of each token. Then, the word representations will be passed to the BiLSTM layer that includes the forward LSTM and backward LSTM, and returns a sequence through exploiting both left and right context information. The outputs of the BiLSTM network are input into the CRF layer. Finally, in the CRF layer, the NE tags will be decoded and output. 

#### 3.2.1. LSTM Layer

RNN, applied to sequential data, is a typical neural network model that is an extension of the traditional feedforward neural network. An RNN contains a recurrent hidden state whose activation of the hidden state depends on the activation of the previous time. However, the gradient disappearance and gradient explosion make the model difficult to deal with long text.

**Figure 4:**
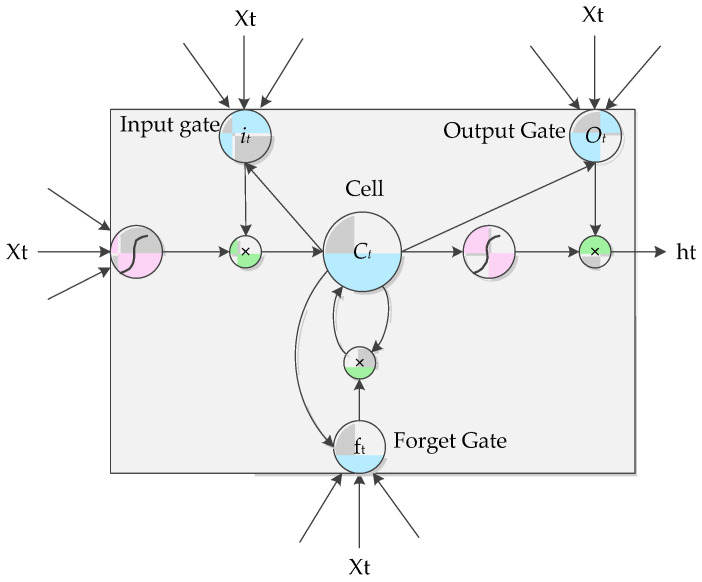
Architecture of the LSTM memory.

LSTMs incorporate a gated memory mechanism to effectively alleviate the long-term dependency limitation [40]. As shown in Figure 4, an LSTM unit includes three gates, an input gate i, a forget gate f and an output gate o. These gates contain a sigmoid neural net layer and a pointwise multiplication operation and are incorporated to optionally remove or add information.

At each step time t, the outputs of the LSTM are iterated to compute by the following equations

(4) $$i_{t}=\sigma (W_{ix}x_{t}+W_{ih}h_{t-1}+W_{ic}c_{t-1}+b_{i}),$$

(5) $$h_{t}=o_{t}⊗tan\;h\left(c_{t}\right).$$

where σ means the sigmoid activation function, ⊗ denotes the elementwise multiplication, and $x_{t}$ is the input vector. Ws with different subscripts represents the weight matrices of the input $x_{t}$, the output $o_{t}$, memory cell $c_{t}$ and hidden state $h_{t}$ respectively; b is the bias matrix for three gates; $i_{t}$, $f_{t}$ and $o_{t}$ are, respectively, the input gate, forget gate and output gate vector at time step t, all of which have the same size as the memory cell vector $c_{t}$ and the hidden vector $h_{t}$.

The task of cultural relics entity recognition can be modeled as sequence labeling task by the deep learning method. There are many long sentences in our data whose focused semantic features can be formed by the characters before and after a long distance, and each cultural relic entity mentioned in the text sequence can rely on the long-distance information text. The bidirectional long and short-term memory (BiLSTM) learns the output weights of the previous moment and the input of each sequence at the current time. Additionally, the past (backward) and future (forward) information of the sentence sequences can be captured simultaneously by the forward network and backward network in the BiLSTM, thus obtaining the context information in the process of sentence sequence modeling. Therefore, this approach is utilized to capture all the information during long-sentence sequence modeling [41]. Based on these characteristics of the LSTM, we utilize the BiLSTM to extract the long-distance dependences of the cultural relic entity.

#### 3.2.2. CRF Layer 

The output of LSTM is the predicted score for each tag, so that we can get the predicted label for each unit in a sentence [42]. However, in the named-entity recognition task, there is no guarantee that the predicted label will predict correctly every time and independent classification is inadequate because the context of sentences in the text has many tagging constraints. When we tag each character individually, the sequences have their own limitations. For example, logically, the tag “I-Name” cannot follow behind the tag “B-LOC”. It is always necessary to consider the correlations between sequential tags and model the dependencies between the output tags [43,44]. CRFs is a discriminant probabilistic undirected graph model more concerned with the level of the sentence than with the individual positions, because CRF considers the correlations between labels in neighborhoods [45]. In our task, instead of decoding each label independently, we add the CRFs layer to the BiLSTM layer of the BiLSTM-CRF network and utilize the CRFs to model the output labels jointly. 

The input of the BiLSTM-CRF network is a sequence $X=\left\{x_{1},x_{2},\ldots ,x_{n}\right\}$, where $x_{1}$ is the i-th word of the input vector, and the predicted output tag sequence is $Y=\left\{y_{1},y_{2},\ldots ,y_{n}\right\}$ for the input sequence X. P, sized n∗k, corresponds to the matrix of scores output by the bidirectional LSTM, where k denotes the number of output tags. $P_{i,j}$ represents the score of the j-th tag of the i-th character, and the score is defined as follows

(6) $$s\left(X,y\right)=\overset{n}{\underset{i=0}{\sum }}A_{y_{i},y_{i+1}}+\overset{n}{\underset{i=1}{\sum }}P_{i,y_{i}\;}.$$

where A represents a square matrix of transition scores that is sized K+2, $A_{y_{i}}$ represents the values of a transition from the tag y to tag i. The start and end tags of a sentence are $y_{0}$ and $y_{n}$.

CRF utilizes a series potential functions to estimate the conditional probability distribution P(y|x,w) of the output tag sequence Y given sequence X. The formula is shown below:

(7) $$P\left(y|x,w\right)=\frac{\exp\left(w^{T}\varphi \left(x,y\right)\right)}{Z\left(w,x\right)}$$

where φ(x,y) represents the feature vector, and w is the parameters vector. The cumulative sum of P(y|x,w) over all the possible y is Z(w,x).

A given training set $\left(Y,X\right)=\left\{x_{i},y_{i}\right\},i=1\ldots n$, is used to train the model by maximizing the conditional likelihood

(8) $$w=arg\;max_{w}p\left(Y|X,w\right),$$

Given the input sequence x and the parameters w, trained by the above method, the tag sequence $y^{*}$ that maximizes the model is the final prediction of a trained CRF

(9) $$y^{*}=arg\;max_{y}p\left(y|x,w\right)$$

The CRF uses the Viterbi algorithm, which can effectively solve training and decoding problems to predict the optimal sequence of tags [22]. The CRF layer considers the limitation between sequences and can automatically learn these constraints through model training to make the final entity tag results more effective.

### 3.3. Semi-Supervised Method

In view of the problem of scarce labeled data in the field of cultural relics, we introduce the semi-supervised method in the NER task. Self-training is a classical semi-supervised learning method that improves the size of the training dataset by learning and supplementing from a large amount of unlabeled corpus [46]. However, in the iterative process, if the number of labeled samples in the initial training sample set is too small, wrong labeling may occur and the errors will be gradually amplified through iteration, which eventually produces an accumulation of errors [47]. Therefore, a semi-supervised model will degrade the performance when mistakes reinforce themselves. To reduce this mistake rate, we adopt a relabeled strategy for unlabeled samples to optimize the self- learning algorithm. A similar algorithm is described in [47].

#### 3.3.1. Self-Learning Algorithm

Self-learning techniques progressively utilize the assumptions they obtain from unclassified samples to predict the unclassified data based on the most reliable predictions [48]. The basic assumption of the self-learning model is that the classifier predicts the samples and the samples with high confidence are iteratively correctly classified [49]. For our NER task, we have two sets of L and U, where L is labeled data and U is unlabeled data. The main steps of the improved self-learning algorithm using the relabeled strategy are presented in Algorithm 1.
**Algorithm 1:** The self-learning algorithm using relabeled strategy**Input:**L is the set of labeled data.    U is the set of unlabeled data.    M is the base model.    ConLev is the Confidence level. **Output:** Trained classifier.
   **Step 1**: Pretrain the model M with labeled data L and obtain the pre-trained model $M^{\prime }$.**Repeat:**   **Step 2**: Predict the unlabeled data U using $M^{\prime }$.   **Step 3**: Select the instances with the predicted probability more than ConLev per iteration ($U^{\prime }$) using relabeled strategy.   **Step 4**: Expand L with $U^{\prime }$ i.e., $L+U^{\prime }\to L$ and remove from *U*.   **Step 5**: Train the model $M^{\prime }$ with *L*.**Until:** some stopping criterion is met or *U* is empty.

Initially, a small amount of labeled data L are selected randomly to pretrain the base model M and obtain the pre-trained model $M^{\prime }$. Then, the pretrained model $M^{\prime }$ is used to predict the labels of the unlabeled data U to label pseudo-labels. The relabeled strategy is used to select the instances with a prediction probability higher than the predetermined confidence level ConLev to generate a set of $U^{\prime }$. Here, the ConLev is confidence level, which is a specific threshold considered sufficiently reliable. These instances are subsequently added to the initial training set L to expand the training set L iteratively (i.e., $L+U^{\prime }\to L$) and increase their efficiency and robustness. Meanwhile, remove $U^{\prime }$ from U. The model $M^{\prime }$ is re-trained using the new expanded training set L until some stopping criterion is met or U is empty. 

#### 3.3.2. Relabeled Strategy

We get the pretrained model $M^{\prime }$ through the first step of Algorithm 1. Next, $M^{\prime }$ is used to train the unlabeled data U. this process is divided into two steps. First of all, after unlabeled data pre-trained by the LSTM classifier in $M^{\prime }$, a category labels and corresponding probability are attained, if the probability is higher than the preset threshold, the pre-label is updated to the word. Then, the word that gets the updated label is input into the CRF model as a feature for prediction. If the predicted result probability is greater than the preset threshold, the labeling result is considered reliable. The sample labeled reliable label is added to the confidence result set $U^{\prime }$. After obtaining the prediction probability through the LSTM model and CRF model, the samples are labeled once respectively. We named this method the relabeled strategy, which is applied to improve the confidence of unlabeled data in addressing the errors that are gradually amplified through iteration. 

## 4. Experimental Results

### 4.1. Data Preprocessing and Annotation

In the preprocessing phase, we collected data from Chinese social media and the Chinese online museum and perform the data preprocessing. Then, we randomly selected a subset of the processed data for data annotation. The remaining large amount of unlabeled data were utilized as the text corpus for semi-supervised training. 

#### 4.1.1. Data Sets 

We collect data used in our experiment from the national museum of China online (http://www.chnmuseum.cn/). The national museum of China is the highest institution for the collection, research, display and interpretation of representative materials that can fully reflect the outstanding traditional Chinese culture, revolutionary culture and advanced socialist culture.

The national museum of China has a collection of more than 1.4 million pieces, covering ancient cultural relics, modern and contemporary cultural relics, books and rare books, works of art and other categories. Among them, there are 815,000 pieces (sets) of ancient cultural relics, 340,000 pieces (sets) of modern cultural relics, over 240,000 pieces (sets) of ancient books, and nearly 6,000 pieces (sets) of first-class cultural relics. In recent years, especially since the 19th national congress of the communist party of China (CPC), the national museum of China has intensified its collection of representative evidence of revolutionary culture and contemporary advanced culture, openly collecting cultural relics from the public, and collecting, on average, approximately 50 sets of ancient relics and over 1000 sets of modern and contemporary cultural relics, objects and works of art every year.

#### 4.1.2. Data Preprocessing 

Online museums display a wealth of text about cultural relics. In this study, we utilize the online cultural relics text as the data resource. We perform preprocessing on the original data. Irrelevant contents are removed, such as html tags and invalid characters. The contents that are shorter than ten characters are filtered out. The difference between Chinese and English is that there are no spaces between the words; thus, word segmentation is performed to separate each word in a sentence. We utilized pyltp, an open source product of the language technology platform (LTP) developed by the social computing and information retrieval research center of Harbin Institute of Technology, providing users with efficient and accurate Chinese natural language processing cloud services. Pyltp provides word segmentation, part of speech tagging, etc. 

In this study, we employed the List of National Cultural Relics Collection (LNCRC) (http://gl.sach.gov.cn/collection-of-cultural-relics/index.html), a website for collecting information about cultural relics, in which the information of cultural relics covers the state-owned museums in 31 provinces, autonomous regions and municipalities. Specifically, LNCRC includes 346,1300 cultural relics, including cultural relic name, cultural relic dynasty, museum collection, etc. We extracted cultural relic information from LNCRC as a supplementary dictionary so that the cultural relic entries matching the knowledge base could be extracted and divided into single words automatically. 

#### 4.1.3. Data Annotation

Data annotation was performed after data preprocessing. We randomly chose a small subset from the preprocessing corpus to annotate and utilize the “BIO” tagging formalism to label entities. The “B” denotes the beginning of an entity, the “I” denotes the continuity of an entity, and “O” represents all other characters. To achieve a professional annotation result, we developed annotation standards in advance and recruited two experts in the field of cultural relics to label entity boundaries and types, and another expert was recruited to check and give the final judgment. The task of NER is to assign a label to each word in a sentence. Take as an example part of a sentence: “*Three |Sheep |Bronze| Lei| was| unearthed| in| Liu| Jiahe*”. The corresponding label is “*B-N| I-N | I-N | I-N | O | O | O | B–L | I–L*”.

### 4.2. Evaluation

#### 4.2.1. Model Evaluation 

In this study, we adopted precision (P), recall (R), and *F*_1_-score (F1) as the performance evaluation parameters. The values of precision, recall, and *F*_1_-score range between 0 and 1, with higher values indicating better performance [50,51].

#### 4.2.2. Baseline Models

To assess the semi-supervised performance of our proposed method in the task of cultural relics named-entity recognition, we compared our model with the following semi-supervised baseline models:

- A unified model in which semi-supervised learning can learn in-domain unlabeled information by self-training, proposed by Xu et al. [36];
- A semi-supervised learning based on the CRFs model for named-entity recognition, proposed by Liao et al. [37];
- A semi-supervised neural tagging that extended the self-training algorithm proposed by Luan et al. [39];
- A combination framework of LSTM and CRF models to complete our NER task, proposed by Yang et al. [11];
- A semi-supervised algorithm that utilized the classical self-training based on our framework named CSCRNER.

The reasons for choosing those semi-supervised models as our baseline model are as follows. First, NER performance is improved by combining large amounts of unlabeled data. Second, semi-supervised learning algorithms based on CRFs have achieved positive performance in the NER task [52]. Third, semi-supervised algorithms based on graphs are proposed because annotated training data are scarce and their semi-supervised strategies are outstanding in the task of information extraction performance [53]. 

### 4.3. Evaluation Results

#### 4.3.1. Performance Comparison of SCRNER and Semi-Supervised Baseline Methods

Based on the experimental results of the 10-fold cross-validation, we set up epochs 20, 40, 60, 80, and 100 times for our model. After 80 times, the improvement of the model tends to be stable, so we took the results when epochs=80 as the experimental results shown in Table 1, which presents the performance comparison of SCRNER with different semi-supervised baseline models across different cultural relic entity types. As can be observed from Table 1, SCRNER, trained by our experimental data, achieves higher average accuracy and a better average *F*_1_-score in recognition of different entities and the overall. 

In general, the accuracy of the SCRNER model (average = 86.7%) (*t* = −22.732, *p* < 0.01) attains 6.1% improvement over the model of [36] (average = 80.6%) (*t* = −20.489, *p* < 0.01), 3.2% improvement over the model of [37] (average = 83.5%) (*t* = −10.284, *p* < 0.01), 1.6% improvement over the model CSCRNER (average = 85.1%) (*t* = −12.482, *p* < 0.01) and 0.7% improvement over the model of [39] (average = 86.0%) (*t* = −2.693, *p* < 0.05). Meanwhile, the SCRNER model (average = 86.1%) (*t* = −24.791, *p* < 0.01) obtained 6.2% improvement over the model of [36] (average = 79.9%) (*t* = −20.682, *p* < 0.01), 4.3% improvement over the model of [37] (average = 81.8%) (*t* = −12.724, *p* < 0.01), 2.4% improvement over the model CSCRNER (average = 83.7%) (*t* = −10.386, *p* < 0.01) and 1.4% improvement over the model of [39] (average = 84.7%) (*t* = −2.319, *p* < 0.05) in terms of the overall *F*_1_-score. 

#### 4.3.2. Performance Comparison of Percentage of Initial Labeled Data

**Figure 5:**
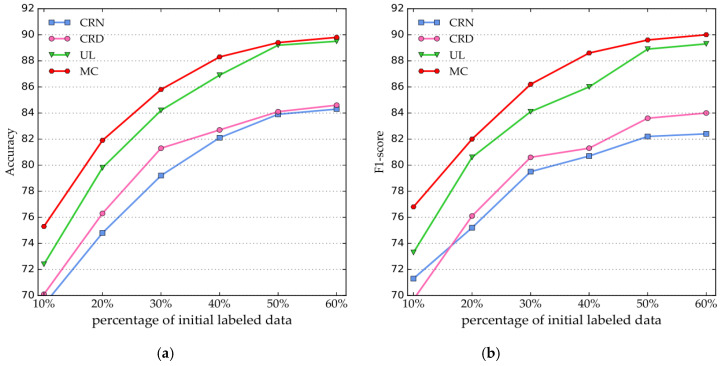
Performance comparison of the percentage of initial labeled data. (**a**) Accuracy curves; (**b**) *F*_1_-score curves.

The performance comparison of the SCRNER model, trained by the initial labeled data with different percentages (from 10% to 60%), is shown in Figure 5. The evaluation results reveal that the higher the proportion of the initial labeled data used for training, the better the performance of SCRNER is for four entities in terms of the accuracy and *F*_1_-score. 

The accuracy of the SCRNER model with 50% of the initial labeled data attains a 14.9% improvement over that with 10% of the initial labeled data, and the F1-score of the SCRNER model with 50% of initial labeled data attains 13.4% improvement over that with 10% of initial labeled data. Furthermore, the accuracy and *F*_1_-score of the SCRNER model increase rapidly with 10%–40% of the initial labeled data, and the two criteria of the SCRNER model tended to be stable with 40%–60% of the initial data. The accuracy and *F*_1_-score of the SCRNER model with 60% of the initial labeled data attained a 0.3% improvement over that with 50% of initial labeled data. 

Combining the experimental results in Figure 5 and in Table 1, we find that 50% of the initial labeled data have achieved a better performance than the baseline models, which proves that our model uses a small amount of labeled data to achieve an effective NER performance.

#### 4.3.3. Performance Comparison of SCNER and Word Representations 

To further verify the performance of the pretrained ELMo model on our proposed approach, we perform comparison experiments of the SCRNER model over different word representations. 

**Figure 6:**
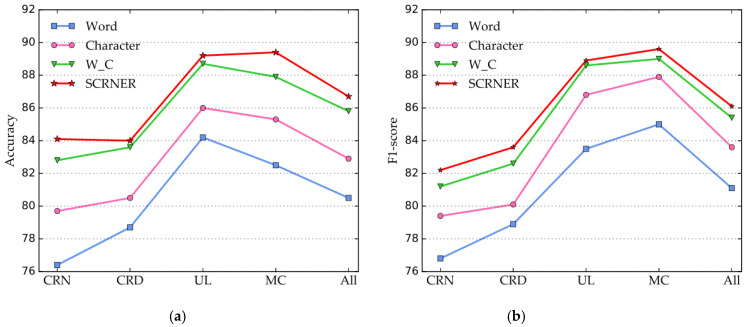
Performance comparison of SCNER and word embeddings. Note: Word, the model that uses word embedding as Word representation; Character, the model that uses character embedding as the word representation; W_C, the model that combines word and character embedding; SCRNER, the model proposed in this study. (**a**) Accuracy curves; (**b**) *F*_1_-score curves.

As shown in Figure 6, the SCRNER model achieves approximately 6.2% (*t* = −8.638, *p* < 0.01), 3.8% (*t* = −9.274, *p* < 0.01), and 0.9% (*t* = − 8.612, *p* < 0.01) higher in terms of accuracy, and 5.0% (t = −4.832, p < 0.05), 2.5% (*t* = −2.968, *p* < 0.01), and 0.7% (*t* = −2.712, *p* < 0.01) higher in terms of the *F*_1_-score of the four entities than the baseline models. The experimental results indicate that the SCRNER model substantially outperforms those baseline models and the use of ELMo for word representations is efficient in our method.

#### 4.3.4. Performance Comparison of SCRNER in Four Entities

**Figure 7:**
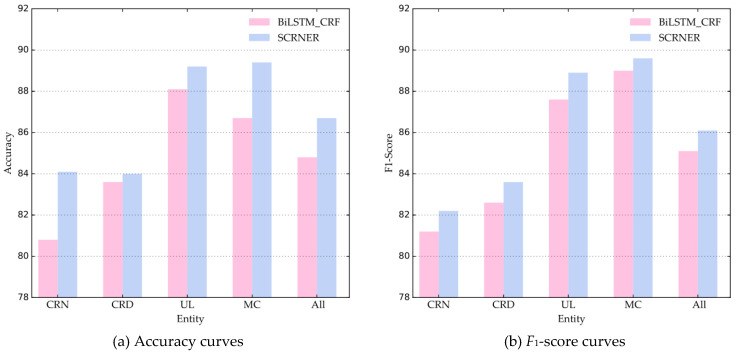
Performance Comparison of BiLSTM-CRF and SCRNER in four entities. Note: BiLSTM_CRF is the framework proposed by Yang H et al. [11].

The performance comparison of BiLSTM-CRF and SCRNER in the four entities is shown in Figure 7. From the perspective of different entity types, the SCRNER model achieves a clear higher performance in terms of the accuracy and *F*_1_-score with the BiLSTM-CRF model across almost all entity types. 

We notice that the BiLSTM-CRF models achieves a relatively lower performance (accuracy 80.8% and *F*_1_-score 81.2%), while our model SCRNER achieves a relatively higher performance (accuracy is 83.9% and *F*_1_-score is 82.2%) in the identification of CRN. Besides, our model SCRNER achieves a higher performance in the identification of CRD, the highest accuracy of 89.4% and the highest *F*_1_-score of 89.6% in the identification of MC. For the recognition task of CRD, UL and MC entities, our model has achieved a good effect. The reason for this may be that the contextual characteristics of these entities are relatively obvious in the text, and ELMo can capture the contextual information of the sentence. Although CRN entity recognition results are lower than those of the other three, accuracy and *F*_1_-score are respectively higher than the BiLSTM-CRF model. As a whole, our model is effective in the task of identifying entities of cultural relics.

## 5. Discussion 

To verify the effectiveness of our proposed model, sufficient experiments are designed to compare the performance of our model with different semi-supervised baseline models. The experimental results demonstrate that our model has a higher performance than the classical self-learning method, which proves the effectiveness of our self-learning method of relabeled strategy. For the overall performance, the experimental results prove that our proposed method achieves high accuracy and an effective F1-score in the other three entity recognition tasks in the field of cultural relics, which verifies that our proposed model is suitable for Chinese online museum data. Moreover, we compare our model based on ELMo with the different word expressions to assess the ELMo performance of our proposed method. The experimental results verify that the effectiveness of the ELMo performance is better than that of word, character and combinations of word and character embeddings, which shows that ELMo captures the internal structure of sentences and generates word representations dynamically based on the context as an effective approach for named-entity recognition in the domain of cultural relics. In addition, the experiments of the performance comparison of the percentage of initial labeled data are designed and implemented. From the experimental results, we find that our model made good use of less initialization annotation data, which proves that our method is applicable to less annotation data in the field of cultural relics.

## 6. Conclusions and Future Work 

Cultural relics named-entity recognition of online cultural relics information is an essential part of entity information extraction for natural language processing. Nevertheless, there is a lack of labeled data in the field of cultural relics, and it is laborious and expensive to label data for deep supervised learning methods to recognize the entities. Moreover, cultural relic entities are complex and diverse, and the boundaries of words are vague. To address these issues, we design an effective semi-supervised deep learning framework based on BiLSTM-CRF, which utilizes the relabeled strategy to select samples of high confidence to train the next iteration. We pretrain the ELMo model as the contextual word embedding to capture the features of the focal character and the contextual information of the related word. Experimental results indicate that our proposed model using limited labeled data outperforms the compared baseline approaches in terms of accuracy and the *F*_1_-score.

Semi-supervised learning can alleviate the problem of having scarce labeled data in training datasets [54]. Self-training is an effective and simple algorithm in semi-supervision. A classifier is pre-trained by the existing labeled data in a self-training algorithm, hence its limitation is that erroneous initial predictions may iteratively generate incorrectly labeled data when a misclassified sample is added to the original training set [55,56]. Accordingly, tri-training or co-training are also promising in semi-supervised methods and are likely to lead to even better results for our work. 

## Figures and Tables

**Table 1:** Performance comparison of semi-supervised cultural relics named-entity recognition (SCRNER) and semi-supervised baseline methods.

Model	CRN(%)		CRD(%)		UL(%)		MC(%)		All(%)	
	Acc	F1	Acc	F1	Acc	F1	Acc	F1	Acc	F1
Xu et al. [36]	76.7	76.2	78.5	76.8	84.8	83.1	82.5	83.4	80.6	79.9
Liao et.al. [37]	79.2	77.1	81.9	79.5	87.5	84.9	85.2	85.6	83.5	81.8
CSCRNER	82.6	80.3	82.0	80.4	88.3	87.1	87.4	86.8	85.1	83.7
Luan et al. [39]	83.4	81.1	83.6	81.3	88.7	87.6	88.1	88.7	86.0	84.7
SCRNER	84.1	82.2	84.0	83.6	89.2	88.9	89.4	89.6	86.7	86.1

**Note.** Entity, CRN, cultural relics’ name; CRD, cultural relics’ dynasty; UL, Unearthed location; MC Museum collection; ALL, the average value of four entities. Model. CSCRNER, a semi-supervised algorithm utilizing the classical self-training based on our framework; SCRNER, the model proposed in this study.
